# Evolving the Mission: Team Process Improvements in Home-Based Primary Care

**DOI:** 10.1093/geroni/igaf122.053

**Published:** 2025-12-31

**Authors:** Tara Afonso, Brian Green, Todd Paul, Cynthia Knight, Shallee Saquing-Nwodo, Katie Connor, Melissa Meynadasy, Michelle Mlinac

**Affiliations:** VA Boston Health Care System, Boston, Massachusetts, United States; VA Boston Health Care System, Boston, Massachusetts, United States; Jamaica Plain VA Medical Center, Jamaica Plain, Massachusetts, United States; Jamaica Plain VA Medical Center, Jamaica Plain, Massachusetts, United States; Jamaica Plain VA Medical Center, Jamaica Plain, Massachusetts, United States; Jamaica Plain VA Medical Center, Jamaica Plain, Massachusetts, United States; VA Boston Healthcare System, West Roxbury, Massachusetts, United States; VA Boston Healthcare System, Boston, Massachusetts, United States

## Abstract

The Home-Based Primary Care (HBPC) model is designed to be an exemplar of integrated interprofessional care, such that HBPC teams can meet the complex medical and psychosocial needs of homebound older adults. Concern for non-specific or undefined goals, challenging patient encounters, and risk of staff burnout highlighted the need to overhaul how the team engaged patients in collaborative care planning. Aims of this 5-year process improvement work were to 1) Update the structure of the interdisciplinary team treatment planning (IDTP) meeting 2) Identify an overall “mission” driving the individualized longer-term care plan for each patient 3) Align current practices with Age-Friendly Healthcare, driven by the 4M’s. These aims were introduced intermittently over time, as team members mastered individual components, integrated them into routine practice, and gained confidence. This work coincided with rapid geographical expansion of the team and a 39% increase in its census. Roles within the team were de-centralized: the documentation responsibility became shared across team members and meeting structure allowed for any team member to serve as the leader. The team achieved a Level 2 Age-Friendly (Committed to Care Excellence) rating by IHI, and in the first year of implementation 98% of HBPC patients (524 out of an average daily census of 533) received 4M’s care.

